# Three-dimensional finite element analysis of silk protein rod implantation after core decompression for osteonecrosis of the femoral head

**DOI:** 10.1186/s12891-019-2914-4

**Published:** 2019-11-15

**Authors:** Liangta Huang, Feiyan Chen, Siqun Wang, Yibing Wei, Gangyong Huang, Jie Chen, Jingsheng Shi, Rajeev K. Naidu, Jun Xia, Tiger H. Tao

**Affiliations:** 1 Department of Orthopedics, The Fifth People’s Hospital, Fudan University, Shanghai, China; 2 Department of Orthopedics, Huashan Hospital, Fudan University, Shanghai, 200040 China; 3 School of Medicine, University of Sydney, Camperdown, NSW Australia; 4 State Key Laboratory of Transducer Technology, Shanghai Institute of Microsystem and Information Technology, Chinese Academy of Sciences, Shanghai, China

**Keywords:** Silk protein rod, Osteonecrosis, 3D finite element analyses

## Abstract

**Background:**

Several methods are available for the treatment of early-stage osteonecrosis of the femoral head. Core decompression with implantation is a widely-used treatment. However, no single implant is recognized as the most effective way to prevent disease progression. Silk has high strength and resiliency. This study explored the possibility of a strong and resilient silk protein biomaterial as a new alternative implant.

**Methods:**

We investigated the biomechanical properties of the silk protein material by regular compression, torsion, and three-point bending tests. We established three-dimensional finite element models of different degrees of femoral head osteonecrosis following simple core decompression, fibula implantation, porous tantalum rod implantation, and silk protein rod implantation. Finally, we compared the differences in displacement and surface stress under load at the femoral head weight-bearing areas between these models.

**Results:**

The elastic modulus and shear modulus of the silk protein material was 0.49GPa and 0.66GPa, respectively. Three-dimensional finite element analyses demonstrated less displacement and surface stress at the femoral head weight-bearing areas following silk protein rod implantation compared to simple core decompression (*p* < 0.05), regardless of the extent of osteonecrosis. No differences were noted in the surface deformation or surface stress of the femoral head weight-bearing areas following silk protein rod, fibula or tantalum rod implantation (*p* > 0.05).

**Conclusions:**

When compared with simple core decompression, silk protein rod implantation demonstrated less displacement and surface stress at the femoral head weight-bearing area, but more than fibula or tantalum rod implantation. Similar effects on the surface stress of the femoral head between the silk rod, fibula and tantalum rod implantations, combined with additional modifiable properties support the use of silk protein as a suitable biomaterial in osteonecrosis surgery.

## Background

Osteonecrosis of the femoral head is a pathological process that can result from internal or external destruction of the blood supply to the femoral head, accompanied by avascularity, cell death, and cartilage collapse, finally results in deformation and avascular necrosis of subchondral bone [1]. As the disease progresses, the femoral head can develop cystic lesions and collapse of the articular surface, with further progression resulting in osteoarthritis. Conservative, non-operative treatment is only suitable in the early stages of osteonecrosis, and surgical intervention is often required as the disease progresses to prevent further damage to the femoral head and to avoid or delay the potential need for an artificial joint replacement [2–4].

The classic surgical treatment of osteonecrosis of the femoral head is core decompression (8 mm/6 mm drill), which can effectively reduce the pressure on the femoral head, improve local blood circulation and relieve hip pain [5–7]; however, this can increase the risk of postoperative fracture and further collapse of the articular surface due to a lack of mechanical support. Currently, core decompression combined with bone impaction grafting or implantation is the main operative approach used in Steinberg stage I-II and can increase the strength of the femoral head and reduce the risk of articular surface collapse [8–11]. Implantation options include porous tantalum rods, vascularized fibular grafting, and nonvascularized fibular graft, etc. [12, 13]. However, neither provides strong structural support, good bone ingrowth, or an uncomplicated operation. The implant design and material, surgical technique, clinical indication and application, and the clinical characteristics of candidates should all be carefully considered and monitored prior to any procedure [13].

Silk protein has now also been widely used as a medical biomaterial in products such as silk protein fibers, silk protein films, and silk protein sponges [14]. As a biodegradable material, silk protein differs from other more conventional materials, while still demonstrating high strength and resilience due to its unique molecular structure [15]. The rate at which the silk protein wall degrades can be regulated and programmed into the material during its preparation process [14], providing a great advantage for its use as a bone replacement material. Processed silk protein can also be embedded with drugs or cell morphogens, like bone morphogenetic protein-2 (BMP-2), has good biological compatibility, is minimally pro-inflammatory [16], and is easier to design and manufacture in a range of shapes, angles, and dimensions as required.

Currently, silk protein is only used with other materials, like hydroxyapatite, as a composite bone filler. A recent study reported that silk protein could be manufactured as silk screws and inserted into rat models with femoral fractures [17]. These screws demonstrated good efficacy with no screws failing during implantation and no postoperative adverse events or screw displacements. Histological results demonstrated osteoclast activity, suggesting early resorption of the silk screws; and osteogenesis of the surrounding tissues, indicating continuous bone ingrowth; demonstrating the potential application of silk protein as a bone substitute material [18–20].

With advances in manufacturing technology, silk protein can now be produced as stronger and larger silk protein rods [21, 22]. The present study aimed to assess the biomechanical properties of the silk protein materials to understand its mechanical properties better and establish a three-dimensional (3D) finite element model of osteonecrosis of the femoral head. To the best of our knowledge, this is the first study investigating the clinical application of the silk protein rod design in the treatment of osteonecrosis of the femoral head, with comparisons made between models of silk protein rod implantation, fibula implantation, porous tantalum rod implantation, and simple core decompression.

## Methods

### Biomechanical testing

**Fig. 1:**
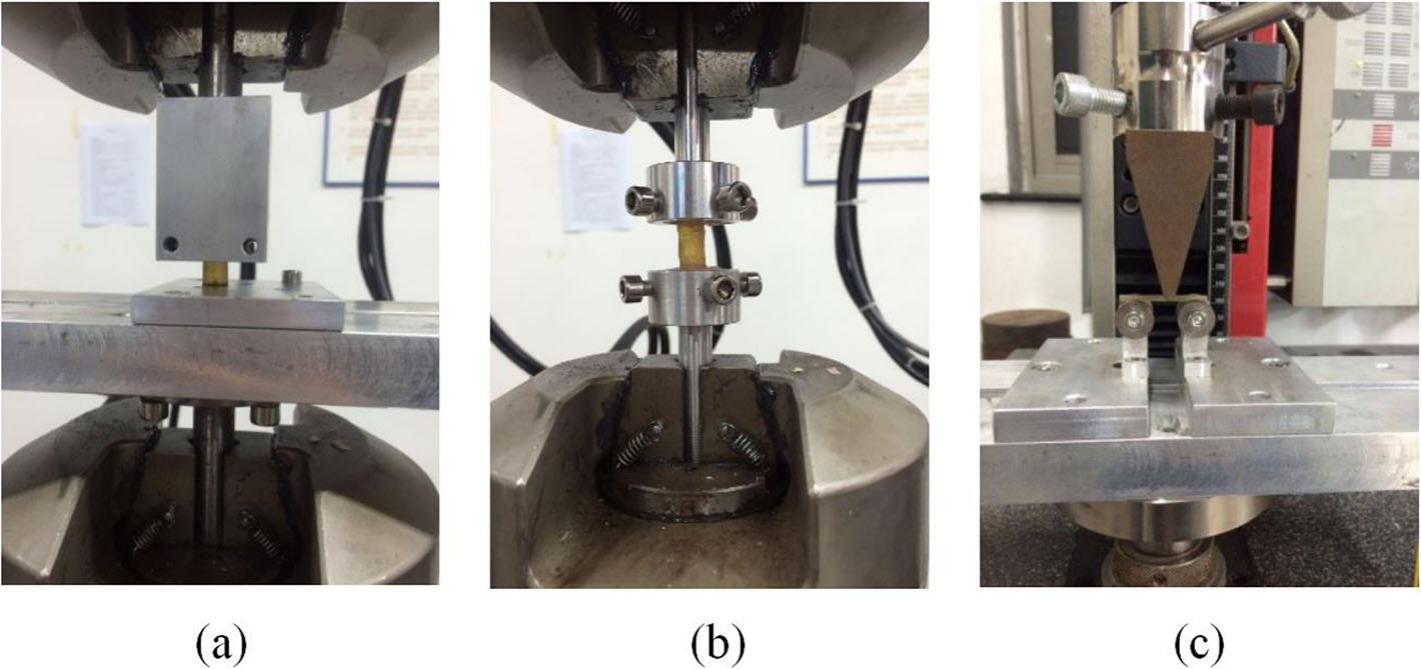
Biomechanical testing process of the silk protein materials. **a** Compression test. **b** Torsion test. **c** Three-point bending test

The biomechanical properties of the harder silk protein materials were tested using six superior segment fibula samples from fresh-frozen human cadavers, with all samples collected from between 2 cm to 12 cm below the head of the fibula. This study was approved by the Ethics Committee of the Huashan Hospital. The participant consent was written and was performed in accordance with the ethical standards of the Declaration of Helsinki of 1964. Three groups were tested: the first group for compression, the second group for torsion and the third group underwent a three-point bending test. The tested substances were fixed at both ends by denture acrylic and denture liquid (mixed in a ratio of 3.5:1). Compression testing and torsion testing were performed using the MTS 370.02 BIONIX machine. The three-point bending test was conducted using the Zwick 2500 N machine (Fig. 1). The compressive force-displacement map, the torsional force-displacement map, and the three-point bending force-displacement map are obtained respectively, and the data are processed by the formula to obtain the elastic modulus and the shear modulus. The Elastic modulus (E) and the Shear modulus (G) were calculated using the following formulae:

$$ Elastic\ Modulus\ (E)=\frac{Tensile\ strength\ \left(\sigma \right)}{Tensile\ strain\ \left(\varepsilon \right)} $$

$$ Shear\ Modulus\ (G)=\frac{Shear\ stress}{Shear\ strain}=\frac{\left(\frac{F}{A}\right)}{\left(\frac{x}{y}\right)} $$

### Three-dimensional finite element analysis

This second stage of the experiment was divided into four groups: the silk protein rod implantation group; the fibula implantation group; the tantalum rod implantation group; and the simple core decompression group was chosen as a control comparison. We established a 3D finite element model of the normal proximal human femur to visualize the distribution of stresses and the overall strength of a normal femur. A healthy 30-year-old male volunteer (normal reference) underwent pelvic and femoral plain radiography to exclude any pathology or abnormality of his proximal femur. 256-slice spiral computed tomography (CT) images were obtained from 5 cm above the upper margin of the acetabulum to 7 cm above the femoral condyle. The data was inputted into a computer-modeling program (Simpleware 2.0 software) to build the 3D finite element model. The original CT images were scanned and processed by a computer-imaging program (Scan IP software), a filter operation performed to remove any noise and a mesh segmentation model created using the CT grayscale images. This model was then reduced to a thickness of 3 mm to acquire a cancellous bone model using a computer-aided design program (Geomagic Studio 11 software). A cortical bone model was obtained by subtracting the cancellous bone model from the full femur model. The resolution of these mesh models was 0.8.

The cortical and cancellous bone materials tested were assumed to be ideal elastoplastic models, and therefore, their known elastic modulus and Poisson’s ratios were assumed for subsequent calculations (Table 1) [23]. The distal femur was securely fastened to limit any directional movements at the bottom of the femur specimens, allowing the fixed models to endure the loads used during testing. In homogenous materials, the center of mass represents the geometric center, and therefore, we approximated the center of the femoral head to correspond to the center of a sphere (Ω) using Geomagic software. A model cone was used to simulate the area of osteonecrosis in the 3D models, with the vertex of the cone directed towards the center of the femoral head. We compared the effectiveness of silk protein rod implantation on different areas of femoral head osteonecrosis, assuming three different cone angles of 60°, 90°, and 120°. The different cone areas were established using ScanIP software.

**Table 1:** Material types of femoral models tested and their assigned parameters

Material structure	Elastic modulus (Mpa)	Poisson’s ratio
Cortical bone	17,000	0.3
Spongy bone	700	0.4

**Fig. 2:**
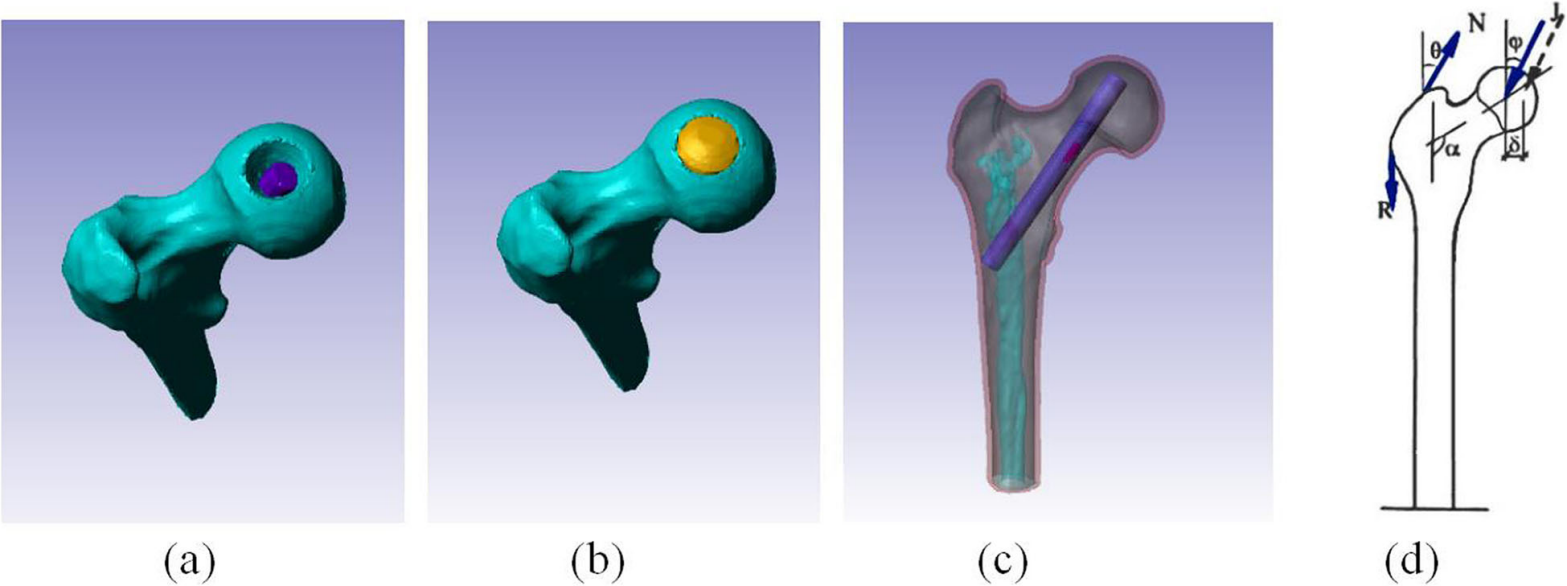
Silk protein rod implantation procedure. **a** Silk protein rod implantation post-core decompression. **b** The remaining osteonecrotic space was filled with artificial bone. **c** Completion of silk protein rod implantation. **d** Force loading diagram. Hip contacting force J = 1620 N, abductor force *N* = 1061 N, iliotibial band force *R* = 1720 N, ψ = 24.4°, θ = 29.5°, α = 135°

The four groups were all tested and modeled in the same way: silk protein rod implantation group; fibula implantation group; tantalum rod implantation group; and simple core decompression group. Using the parameters and results obtained from the biomechanical tests, we produced a 3D finite-element model of the silk protein rod implant and imported the data into the ScanCAD software to assemble the 3D model. The top of the silk protein rod was located 5 mm from the edge of the cortical bone in the femoral head, with the remaining osteonecrotic space filled with artificial bone (Fig. 2). The proximal cadaver fibula implant and the tantalum rod implant were scanned using 256-slice spiral CT, and 3D finite element models of the proximal femur after each type of implantation were produced in the same manner. The material assignment and biomechanical test results are shown in Table 2 [24]. Subsequently, we simulated load bearing on the femoral head weight-bearing area while standing stationary. We assumed a hip contacting force of J = 1620 Newtons (N), an abductor force of *N* = 1061 N, and an iliotibial band force of *R* = 1720 N, with the load angles of ψ = 24.4°, θ = 29.5°, α = 135° (Fig. 2d). However, the 3D finite element model was assumed to be an ideal elastoplastic model, and therefore, all the loads were divided by 10 to avoid unrealistic excessive plastic deformation. We measured the displacement and surface stress at 25 points on the load-bearing region of each femoral head surface tested.

**Table 2:** Assigned parameters of the tested materials in the three-dimensional finite element models

Material structure	Elastic modulus (Mpa)	Poisson’s ratio	Yield stress (Mpa)
Cortical bone	17,000	0.3	111
Spongy bone	700	0.4	3.7
Silk protein	500	0.3	60
Fibula	2000	0.3	20
Tantalum rod	186,000	0.3	
Artificial bone	334	0.3	1.85

### Statistical analysis

Results from all 12 models were analyzed using single-factor ANOVA. A *P* value of < 0.5 was regarded as statistically significant. All results were expressed as the mean ± standard deviation.

## Results

### Biomechanical testing

**Fig. 3:**
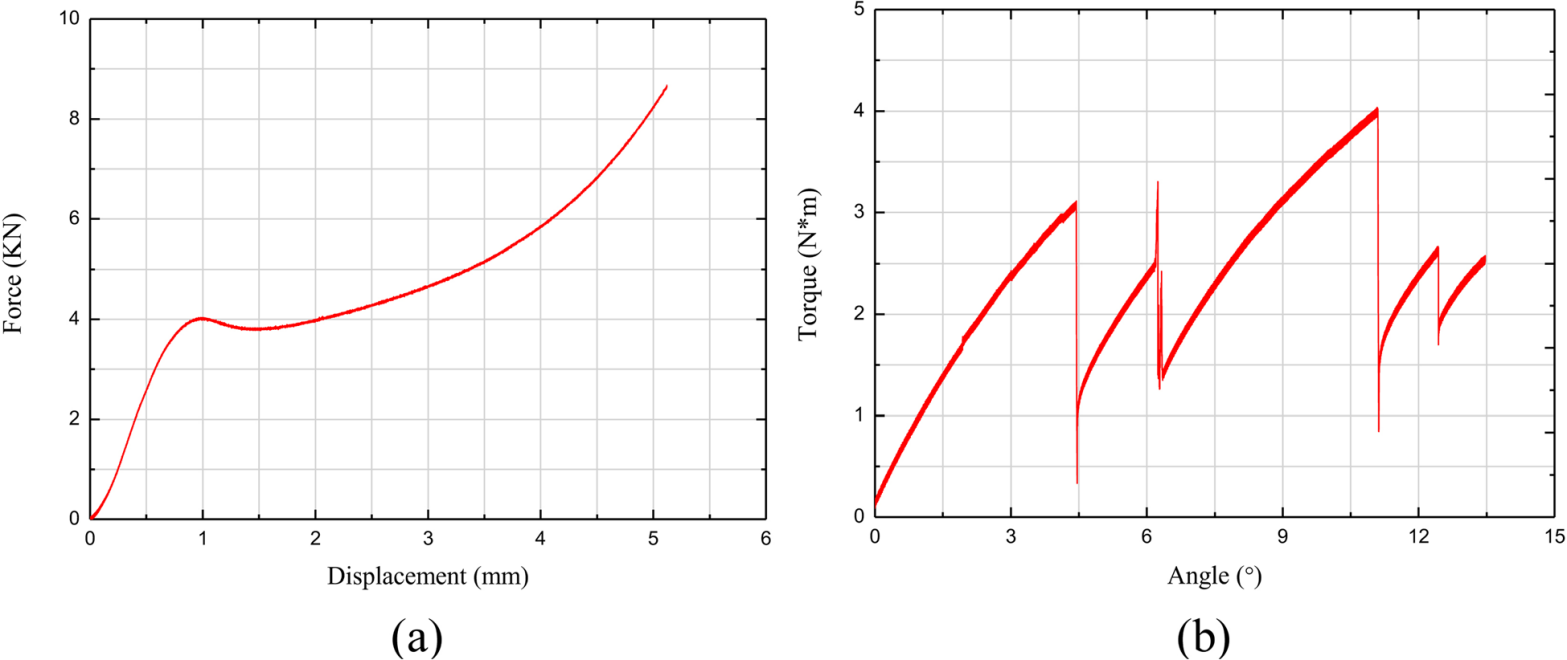
Biomechanical test results of the silk protein material. **a** The displacement and force curve produced by the compression test. **b** The angle and torque curve produced by the torsion test

**Fig. 4:**
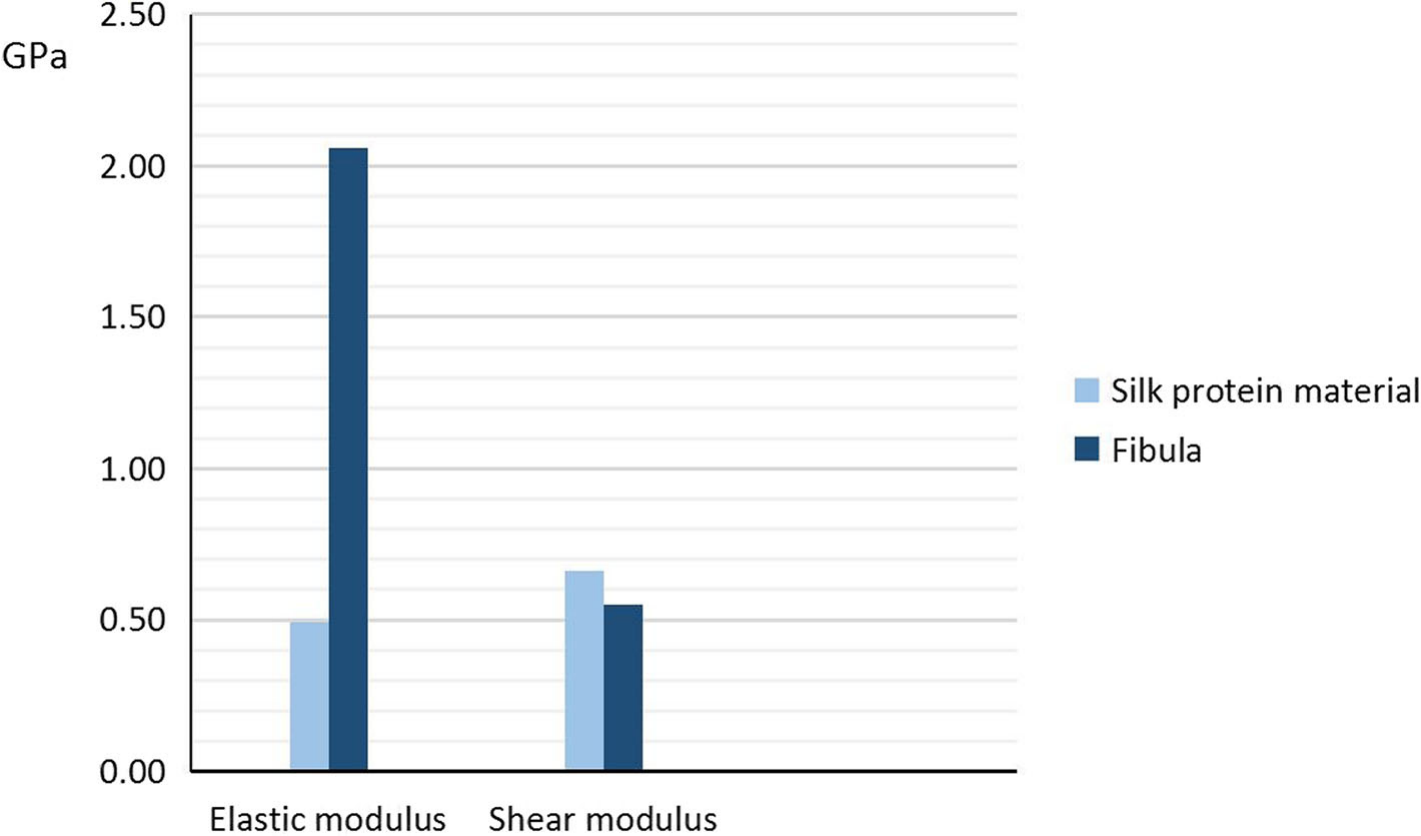
Comparison of elastic modulus and shear modulus of silk protein material and fibula. The average elastic modulus and shear modulus of the silk protein material was 0.49 GPa and 0.66 GPa, respectively. The average elastic modulus and shear modulus of the cadaver fibula sample was 2.06 GPa and 0.55 GPa, respectively. GPa = Gigapascal

The biomechanical results were expressed as a displacement versus force curve. Only the linear portion of the data was used for subsequent analyses. In the compression test, the normal stress was obtained from measuring the axial force, and the normal strain was obtained from measuring the axial displacement. In the torsion test, the maximum shear stress was obtained from measuring the torque, and the maximum shear strain was obtained from calculating the torsional angle. The elastic modulus (E) and the shear modulus (G) were calculated using the stress-strain curve. The elastic modulus of the silk protein material sample 1, sample 2, and sample 3 were 0.15 GPa, 0.83 GPa, and 0.50 GPa, respectively. The elastic modulus of the fibula sample 1 and sample 2 were 1.98 GPa and 2.40 GPa, respectively. The shear modulus of the silk protein material was 0.66 GPa, and for the fibula sample 3 and sample 4 was 0.68 GPa and 0.43 GPa, respectively. Finally, for the silk protein material, the average elastic modulus was 0.49 GPa, and the average shear modulus was 0.66 GPa. For the cadaver fibula samples, the average elastic modulus was 2.06 GPa, and the average shear modulus was 0.55 GPa (Figs. 3, 4).

### Three-dimensional finite element analysis

The displacement of the weight-bearing areas of the femoral head models under the specific loads was measured in the cardinal directions X, Y, Z; with the displacement in each of the three directions designated the finite element units U1, U2, and U3 respectively. The total displacement of the weight-bearing area in the three directions, X, Y, and Z, was calculated as the total vector ‘U’. As the finite element models in this study were all tested under three specific loads; hip contacting force (J), abductor force (N) and iliotibial band force (R); the total displacement (U) of every model tested was measured. Single-factor ANOVA was used for statistical analyses.

### Displacement of the weight-bearing areas of the three-dimensional finite element models of the femoral heads

**Fig. 5:**
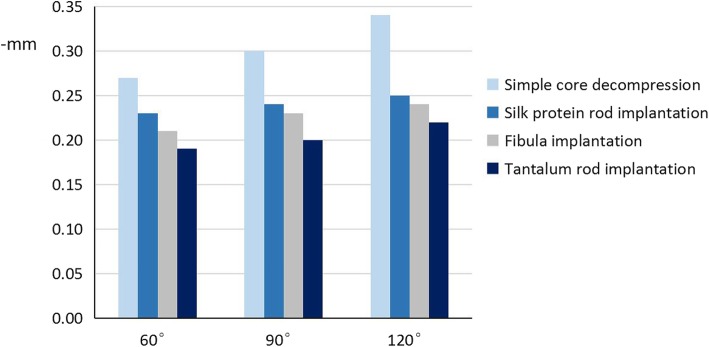
The mean displacement of the weight-bearing areas of the femoral head models according to the different ranges of osteonecrosis and treatments

The displacement of the weight-bearing areas of the different cone angles of the 3D finite element models of all the different femoral head implant options is illustrated in Table 3 and Fig. 5.

**Table 3:** The mean displacement of the weight-bearing areas of the femoral head three-dimensional finite element models (−mm)

Treatment	Osteonecrosis range		
	60°	90°	120°
Simple core decompression	0.27	0.30	0.34
Silk protein rod implantation	0.23	0.24	0.25
Fibula implantation	0.21	0.23	0.24
Tantalum rod implantation	0.19	0.20	0.22

### Mean displacement: the 60° cone area model of osteonecrosis

Pairwise comparison of the 60^o^ cone model showed that the collapse value of the weight-bearing area of the femoral head following silk protein rod implantation (M_sp_) was significantly lower than that following simple core decompression (M_scd_) (M_sp_ = 0.23 ± 0.001, M_scd_ = 0.27 ± 0.001, *p* < 0.05). The amount of displacement of the weight-bearing area after silk protein rod implantation was significantly higher than that following both fibula implantation (M_f_) and tantalum rod implantation (M_tr_) (M_sp_ = 0.23 ± 0.001, M_f_ = 0.21 ± 0.001, *p* < 0.05 and M_sp_ = 0.23 ± 0.001, M_tr_ = 0.19 ± 0.001, *p* < 0.05) (Fig. 5).

### Mean displacement: the 90° cone area model of osteonecrosis

Pairwise comparison of the 90^o^ cone model showed that the collapse value of the weight-bearing area following silk protein rod implantation was significantly lower than that following simple core decompression (M_sp_ = 0.24 ± 0.001, M_scd_ = 0.30 ± 0.001, *p* < 0.05). The amount of displacement of the weight-bearing area after silk protein rod implantation was statistically significantly higher than that following both fibula implantation and tantalum rod implantation (M_sp_ = 0.24 ± 0.001, M_f_ = 0.23 ± 0.001, *p* < 0.05 and M_sp_ = 0.24 ± 0.001, M_tr_ = 0.20 ± 0.001, *p* < 0.05) (Fig. 5).

### Mean displacement: the 120° cone area model of osteonecrosis

Pairwise comparison of the 120^o^ cone model showed that the collapse value of the weight-bearing area following silk protein rod implantation was significantly lower than that following simple core decompression (M_sp_ = 0.25 ± 0.001, M_scd_ = 0.34 ± 0.001, *p* < 0.05). The amount of displacement of the weight-bearing area after silk protein rod implantation was significantly higher than that following both fibula implantation and tantalum rod implantation (M_sp_ = 0.25 ± 0.001, M_f_ = 0.24 ± 0.001, *p* < 0.05 and M_sp_ = 0.25 ± 0.001, M_tr_ = 0.22 ± 0.001, *p* < 0.05) (Fig. 5).

### Von Mises distribution on the surface of the weight-bearing area of the femoral head in the three-dimensional finite element models

**Fig. 6:**
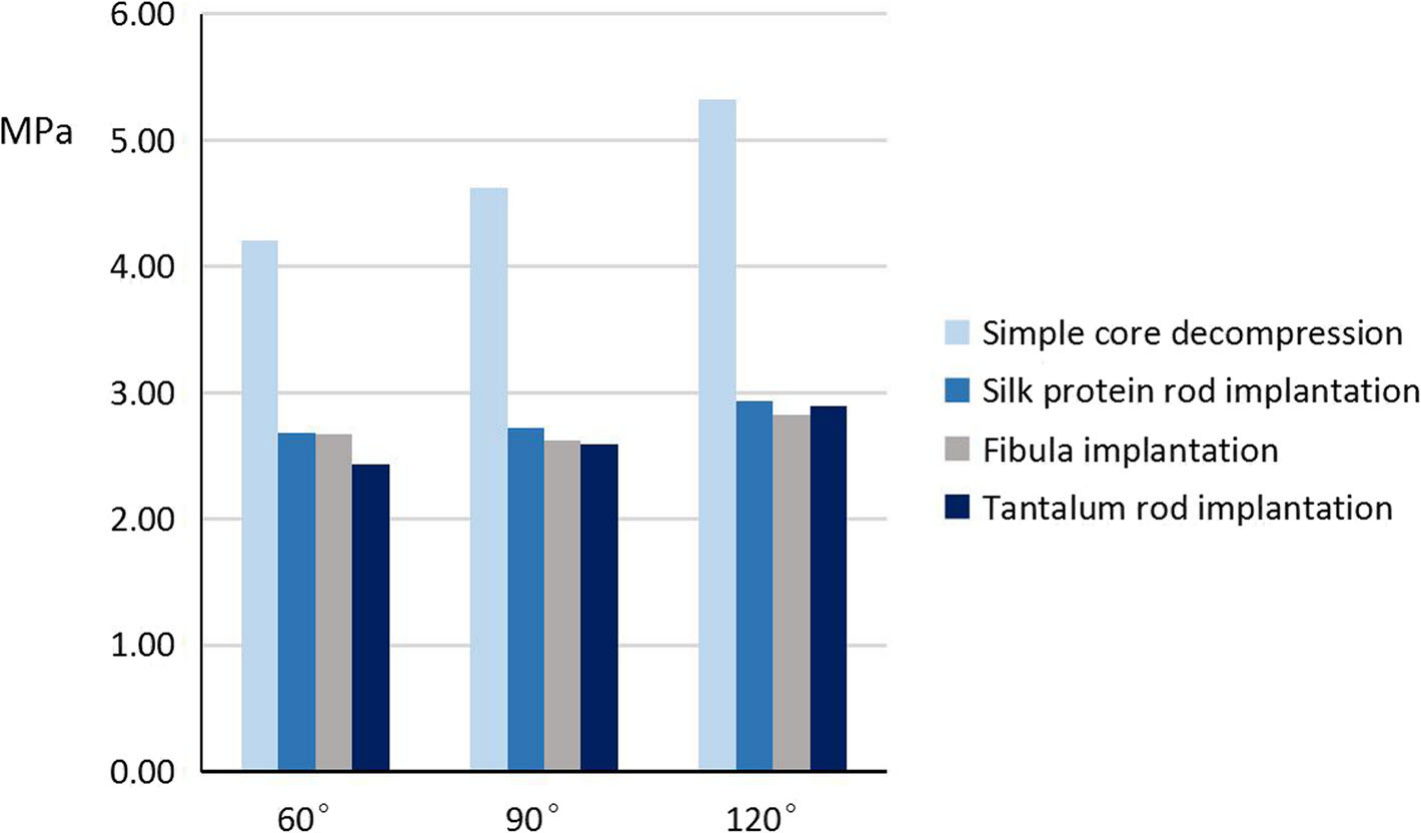
Von Mises distribution on the surface of the weight-bearing areas of the femoral head three-dimensional finite element models

The results of the von Mises distribution on the surface of the weight-bearing areas of the femoral heads in the 3D finite element models based on cone area and treatment option were calculated (Table 4 and Fig. 6).

**Table 4:** von Mises distribution on the surface of the weight-bearing areas of the femoral head three-dimensional finite element models (MPa)

Treatment	Osteonecrosis range		
	60°	90°	120°
Simple core decompression	4.21	4.62	5.32
Silk protein rod implantation	2.68	2.72	2.93
Fibula implantation	2.67	2.62	2.82
Tantalum rod implantation	2.43	2.59	2.89

### Von Mises distribution: the 60° cone area model of osteonecrosis

Pairwise comparison of the 60^o^ cone model showed that the von Mises distribution of the weight-bearing area of the femoral head following silk protein rod implantation was significantly lower than that following simple core decompression (M_sp_ = 2.68 ± 0.26, M_scd_ = 4.21 ± 0.599, *p* < 0.05). The von Mises distribution of the weight-bearing area after silk protein rod implantation was significantly higher than that following tantalum rod implantation (M_sp_ = 0.25 ± 0.001, M_f_ = 0.24 ± 0.001, *p* < 0.05 and M_sp_ = 2.68 ± 0.26, M_tr_ = 2.43 ± 0.295, *p* < 0.05). There were no significant differences in the von Mises distributions between the silk protein rod and the fibula implants (M_sp_ = 2.68 ± 0.26, M_f_ = 2.67 ± 0.413, *p* > 0.05) (Fig. 6).

### Von Mises distribution: the 90° cone area model of osteonecrosis

Pairwise comparison of the 90^o^ cone model showed that the von Mises distribution of the weight-bearing area following silk protein rod implantation was significantly lower than that following simple core decompression (M_sp_ = 2.72 ± 0.347, M_scd_ = 4.62 ± 0.443, *p* < 0.05). The von Mises distribution of the weight-bearing area after silk protein rod implantation was higher than that following both fibula implantation and tantalum rod implantation, but were not significantly different (M_sp_ = 2.72 ± 0.347, M_f_ = 2.62 ± 0.598, *p* > 0.05 and M_sp_ = 2.72 ± 0.347, M_tr_ = 2.59 ± 0.396, *p* > 0.05) (Fig. 6).

### Von Mises distribution: the 120° cone area model of osteonecrosis

Pairwise comparison of the 120^o^ cone model showed that the von Mises distribution of the weight-bearing area following silk protein rod implantation was significantly lower than that following simple core decompression (M_sp_ = 2.93 ± 0.394, M_scd_ = 5.32 ± 0.304, p < 0.05). The von Mises distribution of the weight-bearing area after silk protein rod implantation was similar to that following both fibula implantation and tantalum rod implantation (M_sp_ = 2.93 ± 0.394, M_f_ = 2.82 ± 0.351, *p* > 0.05 and M_sp_ = 2.93 ± 0.394, M_tr_ = 2.89 ± 0.409, *p* > 0.05) (Fig. 6).

## Limitation

Our results should be considered with some caution as some results were based on reported known values and errors in the software and calculations used. The elastic modulus, Poisson’s ratio and yield stress of silk protein rods and fibulas were acquired from previous biomechanical studies; some inherent error resulted from manual operations during 3D finite element studies, such as finite element mesh size and loading sites; and the stress results were obtained using a different method, and may have accumulated some error.

## Discussion

Osteonecrosis of the femoral head is common in China and typically affects young to middle-aged adults, with the potential to cause significant morbidity [1]. Controlling the progression of the disease is important to prevent progression into osteoarthritis, which if severe enough may require an artificial joint replacement. While conservative management is appropriate in the early stages of the disease, implantation following surgical core decompression is the preferred treatment option in more advanced osteonecrosis, including non-vascularised bone grafting, vascularized bone grafting, and tantalum rods, etc. [4]. However, there has been no great success as yet in regards to the ideal implantation biomaterial to use. Silk protein is a new biomaterial that is demonstrating promising results as a potential bone substitute material [25, 26]. The present study assessed the biomechanical properties of the silk protein materials to understand its mechanical properties better and established a 3D finite element model of osteonecrosis of the femoral head. We investigated the clinical application of the silk protein rod design in the treatment of osteonecrosis of the femoral head, with comparisons made between models of silk protein rod implantation, fibula implantation, porous tantalum rod implantation, and simple core decompression.

The first aim of this study was to determine the biomechanical properties of silk protein. Our results of the silk protein demonstrated that the average elastic modulus was 0.49 GPa, and the average shear modulus was 0.66 GPa. For the cadaver fibula samples, the average elastic modulus was 2.06 GPa, and the average shear modulus was 0.55 GPa. Macroscopically, the elastic modulus is an indicator of tensile elasticity. The greater the elastic modulus, the greater the force needed to deform it, indicating greater material stiffness. Our results demonstrated that the silk protein had a lower elastic modulus than the fibula. Despite this reduced stiffness, the silk protein material has been demonstrated to be strong, making an investigation of its application in the treatment of femoral head osteonecrosis worthwhile.

The 3D finite element models produced demonstrated that the amount of surface displacement of the weight-bearing area of the femoral head was less following silk protein rod implantation compared to simple core decompression (*p* < 0.05). This was the case when the osteonecrosis range was 60°, 90°, and 120°, indicating that silk protein rod implantation was better at preventing further collapse of the femoral head. The silk protein did demonstrate greater external displacement than both the fibula and tantalum rod implantation groups, suggesting that the hardness of the silk protein was insufficient to prevent surface deformation. However, there were no differences in the surface stress of the femoral head weight-bearing area, regardless of the range of osteonecrosis, when compared to the fibula or tantalum rod implantations (*p* < 0.05). Surface stress represents the degree of damage to the surface of the weight-bearing region of the femoral head, with our 3D finite element models demonstrating that the silk protein rod can provide good mechanical support under static load-bearing forces, such as when standing.

One limitation of this study was the small sample size. Due to their high production costs, we could only obtain and test a small number of silk protein samples. Unfortunately, one of the silk protein rods was inadvertently damaged due to incorrect use during the torsion test. The samples were also slightly asymmetrical in size and imbedding, potentially resulting in some error when under concentric compression and torsional strain. In the three-point bending test, the diameter of the silk protein samples proved too small, resulting in friction when axial force was applied, potentially affecting the results. Furthermore, the solid denture mix used to fix the silk protein samples during torsion testing had lower ultimate stress than the samples, resulting in it occasionally failing first, which is why the linear torsion results were not fully available. Finally, the irregular geometrical characteristics of the fibula, which we assumed to be approximately elliptical in cross-section, caused a minor error during data processing. Overall, the results of our biomechanical testing, in conjunction with known reference values, were adequate to conduct 3D finite element analyses.

Silk protein has been demonstrated to have good biocompatibility and clinical applications. Although silk protein rod implantation did not prove to be superior to fibula or tantalum rod implantation in preventing further collapse of the femoral head after decompression, it still has the potential for clinical application in osteonecrosis treatment as it can be strengthened, degraded and embedded with bone morphogenic proteins [27, 28]. The silk protein samples used in our biomechanical tests were semi-finished, and not the hardest finished product. The primary objective of our study was to understand the biomechanical properties of silk protein rods and provide a reference for additional improvement of silk protein rods. Therefore, further investigations into silk protein rods as a potential treatment option for early-stage osteonecrosis of the femoral head are worthwhile.

Future research would aim to increase the hardness of the silk protein rod by decreasing the molecular weight of the silk protein constituents. We would propose adding a porous agent to the preparation to roughen the surface and produce a more porous structure, which would produce a design closer to the ideal implant material. A roughened surface increases friction and enhances the stability of the silk rod after implantation, and a more porous structure can better promote new bone ingrowth and embed bone morphogenic proteins and other cytokines.

## Conclusions

The primary objective of our study was to understand the biomechanical properties of silk protein rods and provide a reference for additional improvement of silk protein rods. Silk protein rod implantation demonstrated less displacement and surface stress at the femoral head weight-bearing area compared with simple core decompression. Silk protein holds great potential as an implant material after core decompression to treat early osteonecrosis of the femoral head.

## Data Availability

The datasets used and analyzed during the current study available from the corresponding author on reasonable request.

## Abbreviations

3D: Three-dimensional
BMP-2: Bone morphogenetic protein-2
CT: Computed tomography
